# Patient engagement pilot for uncontrolled hypertension: implications for quality, safety, and population health

**DOI:** 10.3389/frhs.2025.1474634

**Published:** 2025-04-16

**Authors:** Molly L. Daughtry, Kristen E. Miller, David Brennan, Joseph B. Brodine

**Affiliations:** ^1^ Care Transformation Organization, MedStar Health, Columbia, MD, United States; ^2^ Center for Diagnostic Systems Safety, MedStar Health, Columbia, MD, United States; ^3^ School of Medicine, Georgetown University, Washington, DC, United States; ^4^ MedStar Institute for Innovation, Washington, DC, United States

**Keywords:** patient-generated health data, digital health, patient engagement, quality and safety, value-based care

## Abstract

**Introduction:**

Traditional methods of hypertension management often fall short in ensuring timely intervention and sustained patient engagement. This study explores the implementation of a patient-generated health data (PGHD) system using a text-message based platform to enhance patient engagement and improve hypertension control across diverse populations.

**Methods:**

We conducted a patient engagement campaign at MedStar Health within the Maryland Primary Care Program (MDPCP), targeting patients with poorly controlled hypertension across 54 clinics. The intervention utilized the Twistle platform to send automated text messages to patients, encouraging them to submit their home blood pressure readings. Data collection was automated, and the intervention's effectiveness was assessed through engagement metrics and blood pressure control outcomes.

**Results:**

Over a 20-day period, 11,597 patients were targeted, with 9,216 successfully receiving and engaging with the intervention. Of these, 28.5% responded with a blood pressure reading. Follow-up adjustments in patient care plans were made based on 1,209 responses indicating improved control of hypertension. The initiative demonstrated significant improvement in patient engagement and quality of hypertension management.

**Discussion:**

The use of PGHD via text messaging significantly enhanced patient engagement and the management of hypertension, contributing to better quality outcomes and patient safety. This approach proved particularly effective in reaching and impacting patients in underserved communities, where traditional healthcare interactions are often limited. The findings support broader adoption of PGHD interventions in chronic disease management and underscore the potential for digital health tools to transform patient care by actively involving patients in their health management.

## 1 Introduction

Emerging digital technologies provide exciting opportunities for patient-generated health data (PGHD). The Assistant Secretary for Technology Policy/Office of the National Coordinator for Health Information Technology (ASTP/ONC) defines PGHD as health-related data created, recorded, or gathered by patients, family members, or caregivers to help address a specific health concern (1). As healthcare shifts toward patient-centered care, PGHD presents new opportunities to optimize outcomes. The growing availability of data collection tools via consumer devices (e.g., smartphones, wearables) makes it easier to gather and use this data effectively (2). Chronic disease conditions, like hypertension, particularly benefit from PGHD. Continuous monitoring allows for early detection of abnormal trends, enabling timely medical interventions (3–6). This real-time data collection improves the accuracy of diagnosis and treatment plans, enhances patient engagement in their own health management, and ultimately leads to better quality and safety in care delivery.

Hypertension (high blood pressure) is a significant risk factor for heart disease and stroke, two leading causes of mortality in the United States (7–10) and is further cited as the leading single preventable risk factor worldwide for mortality (11). Hypertension control rates in the United States have worsened over the last decade with individuals belonging to racial and ethnic minority groups experiencing notably lower rates of control (12). The burden of hypertension is higher among Black adults (56%) than non-Hispanic white (48%), non-Hispanic Asian (46%), and Hispanic (39%) adults (13, 14). This higher prevalence is attributed to various factors including social determinants of health that impact access to healthy foods, safe places to walk, and practices of discrimination both within and external to the healthcare system. Hypertension-related mortality is four to five times greater for non-Hispanic Black adults when compared to non-Hispanic white adults (15). Hypertension control is the top disparity in primary care within our healthcare system. While there are system-wide supports for following hypertension control guidelines, health system strategies specifically tailored to primary care practices and to the population experiencing lower achievement of target blood pressure are needed (16–18).

Financial penalties within healthcare systems are part of broader efforts designed to encourage healthcare providers to improve their care practices, reduce avoidable hospitalizations, and ultimately lower overall healthcare costs. Accountable Care Organizations, state-level initiatives, and private insurance penalties also play a role in promoting effective chronic disease management through value-based care models. Poor management of chronic conditions like hypertension may result in reduced payments or other financial penalties such as the pay-for-performance (P4P) model (19), financial incentives paid to physicians and/or patients (20, 21), Medicaid incentives for the Prevention of Chronic Diseases (22), and innovative payment models including the Accountable Care Organization (23), patient-centered medical home (24), and the Million Hearts Cardiovascular Disease (CVD) Risk Reduction Model programs (25). Controlling hypertension is a particularly challenging measure given the Centers for Medicare and Medicaid Servies (CMS) quality metric (ID #236: Controlling High Blood Pressure) is determined by the last documented reading at the end of a calendar year (26). The full measure description is “percentage of patients 18–85 years of age who had a diagnosis of essential hypertension starting before and continuing into, or starting during the first 6 months of the measurement period, and whose most recent blood pressure was adequately controlled (<140/90 mmHg) during the measurement period”. Therefore, any number of normal blood pressure readings can be supplanted by a single high reading at the end of the year.

Traditional tactics to address hypertension include resource intensive quality improvement sprints at the end of the year including phone calls and portal messages to request a new reading, aiming to capture patients who are generally well controlled but had a recent elevated blood pressure. Most patients who “failed” the measure were within 10–15 mmHg above the threshold so it is likely that a new reading would be within the normal range. Particular given that home blood pressure monitoring is often more accurate than readings taken in clinical settings because it can provide multiple measurements over time in a familiar and relaxed environment, reducing the influence of white-coat hypertension and other situational factors (27).

The objective of this initiative was to pilot a text-message based patient engagement solution to capture home blood pressure readings for patients with uncontrolled hypertension to accurately update their electronic health record (EHR) with a reading more representative of their baseline measurement. The findings of this study demonstrate the feasibility of an automated approach to improve the accuracy of performance on quality metrics while creating a safety surveillance mechanism. Leveraging PGHD through a patient-centric solution offers insights for workflow improvements to reduce the resource burden of the care team, improve the patient experience, and ultimately improve quality and safety which as a result impacts financial incentives.

## 2 Methods

### 2.1 Design and setting

In December 2023, MedStar Health implemented a pilot automated outreach program within the Maryland Primary Care Program (MDPCP) aimed at addressing uncontrolled hypertension. This study was conducted across 54 clinics participating in MDPCP, which provides care management for approximately 45,000 Medicare B patients. The MDPCP follows a value-based care model that incentivizes clinics based on their performance in cost and quality measures, with each practice being uniquely assessed. This quality improvement project was retrospectively reviewed and approved by the Institutional Review Board (IRB) of MedStar Health Research Institute for the purpose of publication.

### 2.2 Target population

We identified MDPCP patients with hypertension who had inadequately controlled blood pressure (>139/>89 mmHg) or no recorded blood pressure during the 2023 calendar year. Patients meeting these criteria, 18 years of age or older, and English speaking were included in the study. Detailed demographic information, such as age, gender, and comorbidities of the identified patients, was not included in this pilot.

### 2.3 Overview of initiative

Twistle is a patient engagement platform that complies with Health Insurance Portability and Accountability Act (HIPAA) regulations and is adaptable for use in various clinical specialties, procedures, and chronic conditions (28). This versatile platform can send messages and reminders via multiple channels, including smartphone apps (both Apple and Android), text messages (SMS), computer browsers, and traditional landlines using interactive voice response (IVR) or pre-recorded voice messages. Twistle ensures that participants can receive communications regardless of their geographic location, effectively addressing internet and provider access challenges in rural areas (29, 30).

**Figure 1:**
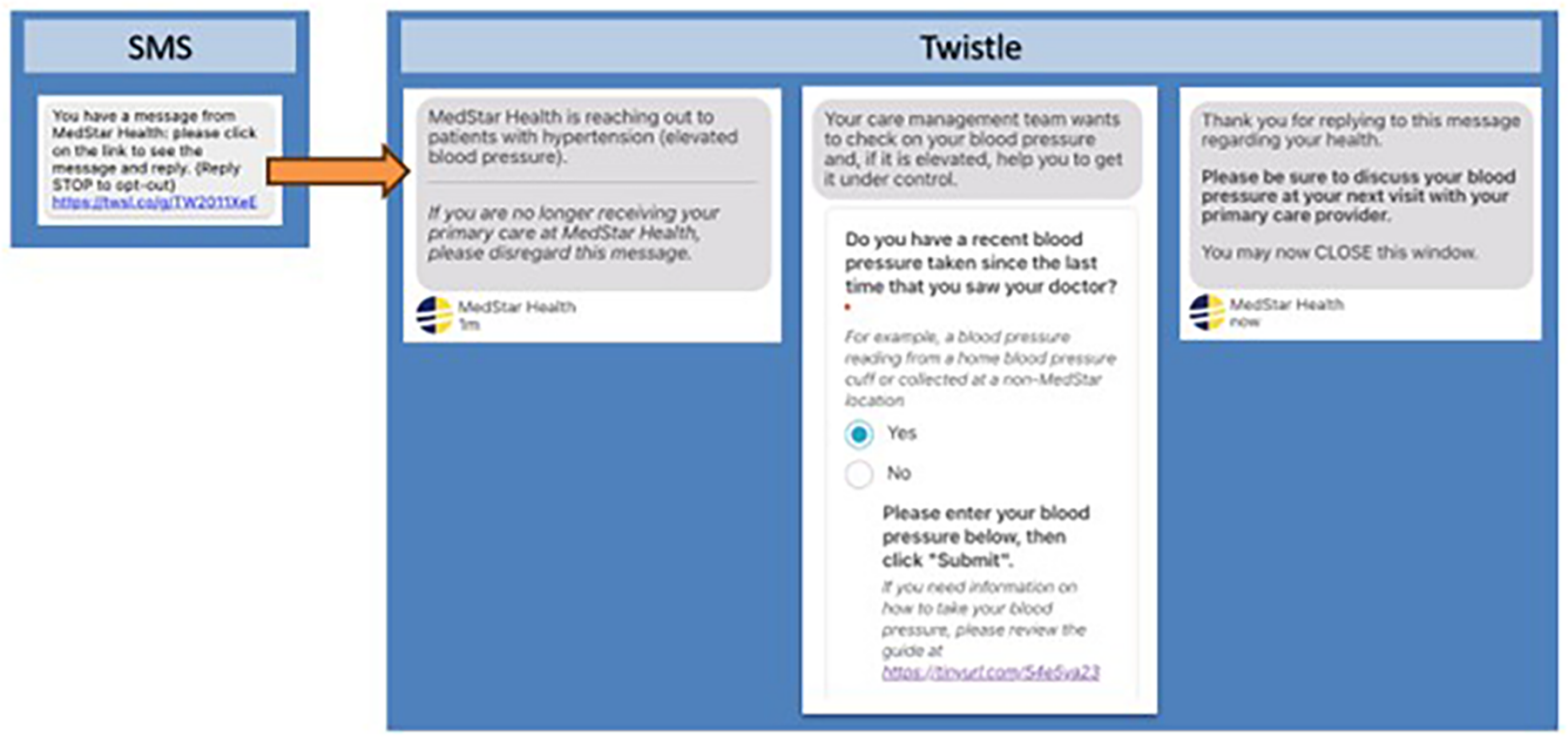
Outreach messages sent via Twistle to eligible patients including initial SMS message, introduction message (once URL is clicked), and data collection form requesting the patient's recent blood pressure.

Over a 20 days period, we sent invitations in ten waves (∼500–2,000 patients per wave) batched by clinic (5–8 clinics per batch). Eligible patients received an automated text message via the Twistle patient engagement platform, inviting them to engage in a scripted chat exchange (Figure 1). We made two follow-up attempts for any patient who did not respond to the initial message (first reminder on Day 2, second reminder on Day 7). Primary care providers were made aware of the program through email and quality/safety meetings. Care Transformation Organization (CTO) team members including nurses, medical assistants, and care managers were responsible for direct follow-up with patients which aligns with their broader role to address quality measures for MDPCP patients including blood pressure, diabetes, and preventative health screening (e.g., mammography, colorectal cancer).

**Figure 2:**
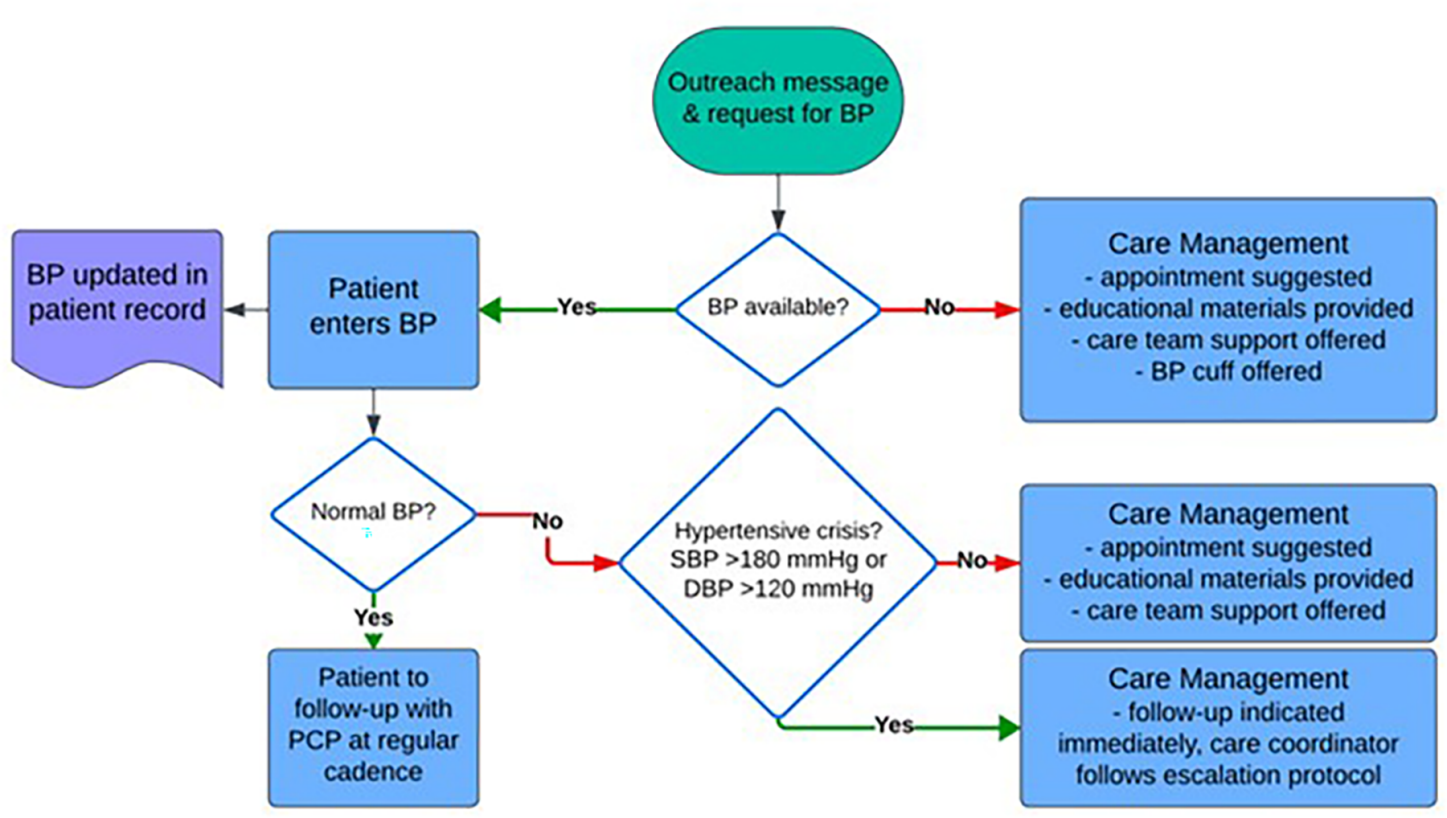
Flow diagram for workflow and documentation process used by care coordinators for the initiative.

The dynamic, algorithmically driven text exchange was designed to capture their recent home blood pressure readings, if available, and provide care management (Figure 2). Patient information, including their responses through Twistle, were populated on a secure dashboard within the Twistle platform. An escalation protocol was used based on the patient's response. Patients also had the option to request additional resources including educational materials about controlling blood pressure, scheduling assistance to see their doctor or another provider at the clinic, support from a care manager to help with controlling blood pressure, and placing a request to receive a blood pressure cuff for home use. Blood pressure readings reported by patients through Twistle were transcribed into the EHR by the CTO team as well as clinical notes reported by the CTO team following outreach to patients.

### 2.4 Outcome measures and statistical analysis

During the pilot period, we tracked the number of patients who successfully engaged with the Twistle outreach defined as received the Twistle link and then opened the link and then submitted a response (primary outcome). We categorized the blood pressure readings into normal (<140/<90 mmHg), uncontrolled (140–180/90–120 mmHg), and hypertensive crisis (>180/>120 mmHg) categories. Additionally, we recorded the number of patients requesting further assistance like education, scheduling, support, or device (secondary outcomes). The statistical analysis for this pilot study focused on basic descriptive statistics to summarize the key outcomes and characteristics of the study population. The analyses will be performed using Excel, and results will be presented as means, standard deviations, medians, interquartile ranges, frequencies, and percentages as appropriate. Rapid thematic analysis was conducted on a sample of clinical notes describing the follow-up by the CTO team.

## 3 Results

### 3.1 Outreach results

**Figure 3:**
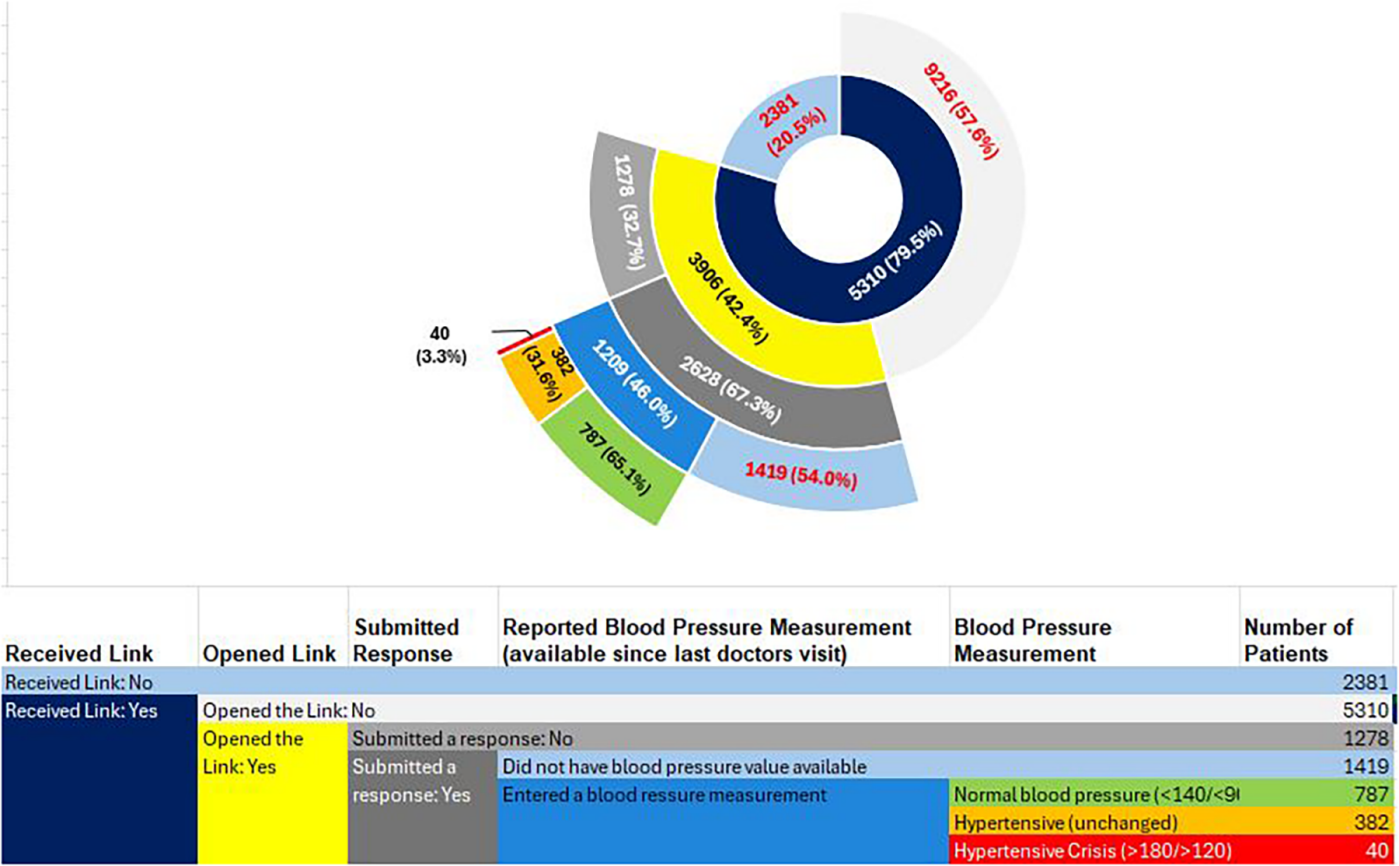
Patient engagement from initial outreach through patient response (reporting most recent blood pressure measurement categorized by severity).

Over the 20-day pilot period, we initiated outreach to 11,597 eligible patients. Accounting for provision errors (inability to load into Twistle platform), SMS errors (non-working phone numbers), and opt-outs, 9,216 patients successfully received the Twistle link, representing 79.5% of the initially eligible patients. Out of 9,216 patients who received the Twistle link, 42.4% opened the link and 28.5% submitted a response to the first prompt “Do you have a recent blood pressure taken since the last time you saw your doctor?”. For patients that selected yes (1,209), 8.5% reported a normal value, 4.1% reported a hypertensive value, and 0.4% reported a hypertensive crisis value (Figure 3).

Of the 2,628 patients who submitted a response, 488 (18.6%) requested additional resources: 321 requested educational materials about controlling blood pressure, 186 requested scheduling assistance to see their doctor or another provider at the clinic, 10 requested support from a care manager to help with controlling blood pressure, and 49 requested a blood pressure cuff for home use. Some patients requested multiple resources resulting in 566 unique requests.

We aimed to determine when patients submitted their responses following the initial outreach and subsequent reminders. After the initial message, 1,582 patients responded, accounting for 60.2% of the total responses. Following the first reminder, sent 2 days later, an additional 720 patients responded, representing 27.4% of the responses. The second reminder, sent 1 week after the initial message, prompted 326 more responses, making up 12.4% of the total.

### 3.2 Patient challenges identified during outreach

We conducted a rapid thematic analysis of clinical notes entered by the quality team following outreach to patients in hypertensive crisis. Key findings highlighted the value of the initiative to serve as a prompt to measure their blood pressure and a reminder of the need to regularly monitor blood pressure at home, challenges with medication adherence (specifically the need to refill prescriptions), and access to a blood pressure home monitor (Table 1).

**Table 1:** Key themes from qualitative analysis of clinical notes describing patient outreach and follow-up for patients self-reporting hypertensive crisis (>180/>120 mmHg).

General theme	Sample text from clinical note
Prompt: measure blood pressure	“Getting the text forced her to take her BP.”
Reminder: regularly monitor blood pressure at home	“Patient was contacted regarding bp reading of 151/101. He stated that he took his bp again and it was 138/88. He] was made aware to keep a log of his daily bp readings and follow up with PCP in the near future.”
Reminder: regular follow-up with PCP	“[Patient stated] a lot has been happening in her life that has prevented her from being seen regularly with her PCP… An appointment was secured for today at 11 a.m.… Patient confirmed that she will attend the appointment.”
Medication adherence: refill medications	“[Patient] stated that she had been out of medication for some time.” “She expressed how appreciative she was for the text because she had run out of her blood pressure medication, didn't have any obvious symptoms…”
Device: access to home blood pressure monitor	“BP cuff ordered.”

## 4 Discussion

There are operationally and clinically significant findings of this initiative. In reviewing the outreach and the data ultimately provided by patients identified with uncontrolled hypertension, 1,209 patient records were updated with a recent blood pressure. From these responses, 797 records were updated to reflect their blood pressure is now adequately controlled, improving the accuracy of the patient's data but also removing them from the percentage defined as uncontrolled per the CMS definition. This in turn, improves quality metrics impacting financial incentives. Perhaps more important to the individual patient, the remaining patients reporting values identified as hypertensive or hypertensive crisis received immediate follow-up to include a broad range of clinical management ranging from education to care team support and assistance scheduling clinical care (e.g., primary care in person or telehealth, urgent care). It is important to highlight that all 40 patients identified as hypertensive crisis received immediate follow-up where a care manager directly reached the patient by phone within 24 h of the patient submitting their blood pressure reading. Immediate follow-up activities included facilitating same-day follow-up with their primary care provider or with an urgent care provider, helping to get medications refilled, and establishing a home blood pressure monitoring regimen. Without this initiative, patients would not have received a prompt to measure or report their blood pressure and may not have been aware of their current health status. The immediate follow-up activities ensure patient safety and may have prevented adverse events such as myocardial infarction or cerebrovascular accident. Clinical notes further demonstrated the intrinsic value of safety embedded in quality improvement.

Separate from the clinical findings, this initiative provides insights into the feasibility of asynchronous automated outreach to patients. PGHD opportunities have developed alongside emerging socio-technical challenges (e.g., workflow integration, patient adoption) which may limit their use (31). This digital outreach pathway was designed to minimize the time and resources required compared to traditional phone call-based methods, providing patients with the flexibility to respond at their convenience. Care managers followed up with patients who requested assistance or reported elevated home blood pressures. As compared to traditional approaches requiring significant human resources, patient engagement solutions like text-message based platforms or those that leverage the patient portal, decrease the manual labor for the initial outreach. These asynchronous approaches may ultimately be more feasible and effective from the perspective of the patient for several reasons. Many people tend to ignore or decline calls from unknown numbers, which makes phone outreach less reliable. Text messages, on the other hand, are generally more accessible and less intrusive, allowing patients to respond at their convenience. This flexibility accommodates varying schedules and reduces the pressure to respond immediately, making it easier for patients to engage with their healthcare on their own time. Additionally, text messages provide a written record that patients can refer back to, enhancing the clarity and retention of important health information. This method also allows for automated, scalable communication, ensuring that a larger number of patients can be reached efficiently.

This initiative yielded crucial insights into optimizing the timing and frequency of text messages, significantly enhancing patient engagement and participation rates. By analyzing patient response rates to the initial message and subsequent reminders, the program demonstrated that a significant portion of patients responded to the first text message. Specifically, 60.2% of responses were received after the initial message, highlighting its effectiveness. Furthermore, the initiative showed that sending a first reminder 2 days after the initial message was effective in generating an additional 27.4% of responses while the second reminder, sent 1 week after the initial message had a smaller impact, generating 12.4% of the responses. This pattern underscores the importance of timely follow-ups to maximize patient engagement in the program and suggests that a well-timed sequence of messages can maximize patient engagement while maintaining a balance between persistence for patients' time. By understanding the optimal intervals for follow-up messages, healthcare providers can better plan and allocate resources, ensuring that outreach efforts are both efficient and effective.

In reviewing the number of patients who ultimately submitted a response in comparison to the number of eligible patients, there are definitely opportunities for improved outreach. In addition to the interval of follow-up, there are additional considerations where further customization could enhance effectiveness while also addressing health equity. Accounting for personal preferences in patient outreach is crucial including message timing, language, method, and tone. The timing of text messages can significantly impact patient engagement. Sending messages at times when patients are most likely to be available and attentive, such as during the evening or on weekends could increase response rates. Tailoring communication to meet the needs and preferences of patients is a crucial step in fostering trust and could impact engagement. Using the patient's preferred language and recognizing cultural nuances can make the message more accessible and demonstrate cultural sensitivity, fostering a stronger connection between the patient and the healthcare provider. Trust is a fundamental element in the relationship between patients and healthcare providers. It plays a critical role in ensuring that patients feel comfortable engaging with their healthcare, sharing sensitive information, and adhering to medical advice. Ensuring that messages clearly identify the sender and convey the legitimacy of the outreach can alleviate concerns about potential scams or spam. Providing recognizable and trustworthy sender information can help patients feel more secure in responding to the messages. Finally, it is essential to consider that some patients may avoid disclosing unfavorable health information, such as high blood pressure readings. This may have contributed to the number of patients who opened the text link but did not submit a response. Creating a supportive and non-judgmental environment within the messaging can encourage honesty and transparency. Emphasizing the importance of accurate health information for better care and offering reassurances about privacy and confidentiality can help patients feel more comfortable sharing their data. More research is required to explore the characteristics of the patients who responded vs. those that did not and identify factors that may have contributed to their engagement.

There were many lessons learned for our organization from this initiative including challenges in transferring PGHD captured through Twistle into the EHR. There was no data pipeline to “write” into the EHR which then required manual updating leading to increased effort and transcription errors. We must also consider transcription errors from the patient data entry with no way to validate the response, relying entirely on the honesty and accuracy of the patient. While the initiative was designed to address quality measures, the resulting outreach from the CTO team led to increased primary care visits which highlights the need to partner and prepare primary care practices.

This pilot demonstrated that patient engagement solutions serve as a force multiplier for existing teams performing manual outreach, significantly enhancing their efficiency, improving quality measures and quality of care, and enhancing patient safety on an individual level. Extending this approach to other health measures, such as A1C lab results for diabetes, mammography, and colorectal cancer screening, could replicate success across diverse healthcare settings, promoting patient-centered care and proactive health management. Beyond data capture, the platform facilitates the sharing of education resources, provision of care management, and inquiries about access to resources like medical devices. By improving patient engagement, these solutions ultimately enhance patient outcomes and contribute to financial gains for healthcare systems.

## Data Availability

The raw data supporting the conclusions of this article will be made available by the authors, without undue reservation.
